# Oxidative and Nitrosative Stress in Atopic Dermatitis and Depression: Similarities in Biomarkers and Pathophysiological Mechanisms

**DOI:** 10.3390/pathophysiology33020039

**Published:** 2026-06-10

**Authors:** Dominika Jabłonka, Stefan Modzelewski, Napoleon Waszkiewicz

**Affiliations:** Department of Psychiatry, Medical University of Bialystok, pl. Wołodyjowskiego 2, 15-272 Białystok, Poland; stefan.modzelewski@sd.umb.edu.pl (S.M.); napoleon.waszkiewicz@umb.edu.pl (N.W.)

**Keywords:** atopic dermatitis, major depressive disorder, oxidative stress, nitrosative stress, redox biomarkers, lipid peroxidation, DNA oxidative damage, antioxidant defense, skin–brain axis, inflammation

## Abstract

Background: Atopic dermatitis is a chronic inflammatory skin disease characterized by epidermal barrier dysfunction and immune dysregulation, whereas major depressive disorder is a common psychiatric condition with a substantial impact on quality of life; increasing attention has been given to oxidative and nitrosative stress as a potential biological link between these disorders. Methods: This narrative review synthesizes current evidence on molecular biomarkers of oxidative and nitrosative stress in AD and MDD and examines shared mechanisms within the skin–brain axis. Results: Across both conditions, studies consistently report increased markers of lipid peroxidation (e.g., malondialdehyde, 4-hydroxynonenal), oxidative DNA damage (8-hydroxy-2′-deoxyguanosine), and nitrosative stress, alongside impaired antioxidant defenses, particularly involving glutathione; these alterations are closely associated with chronic inflammation, cytokine signaling, mitochondrial dysfunction, and dysregulation of neuroimmune and hypothalamic–pituitary–adrenal axis pathways. Conclusions: Although the available evidence is heterogeneous and largely based on cross-sectional studies, limiting causal inference, the findings support a biologically plausible link between AD and depression mediated by shared redox pathways and highlight the need for further longitudinal and mechanistic research.

## 1. Introduction

Atopic dermatitis (AD) is a chronic inflammatory skin disorder marked by epidermal barrier dysfunction and immune dysregulation. AD commonly coexists with other atopic conditions, such as asthma and allergic rhinitis, and is often regarded as an early step in the “atopic march” [1,2,3]. Over the past years, increasing attention has been given to the role of oxidative and nitrosative stress in AD. Excessive production of reactive oxygen and nitrogen species, together with insufficient antioxidant defense, may contribute to keratinocyte injury, barrier impairment, and perpetuation of cutaneous inflammation. In line with this, elevated levels of oxidative damage markers such as malondialdehyde (MDA) and 8-hydroxy-2′-deoxyguanosine (8-OHdG) have been associated with greater disease severity, suggesting that redox imbalance may be involved not only in disease progression but also in its underlying pathophysiology [4,5]. Major depressive disorder (MDD) is a common and disabling psychiatric illness that has also been linked to oxidative–nitrosative imbalance. Patients with MDD show reduced antioxidant capacity together with increased oxidative damage to lipids, proteins, and DNA (deoxyribonucleic acid). These alterations are closely related to neuroinflammation, mitochondrial dysfunction, and dysregulation of the hypothalamic–pituitary–adrenal axis, all of which may contribute to symptom severity and disease progression [6,7]. Importantly, epidemiological studies indicate that individuals with atopic diseases, including AD, are at increased risk of depression. This association supports the concept of a “skin–brain axis,” involving bidirectional interactions between immune, neuroendocrine, and redox pathways [8,9]. Although interest in this topic has grown substantially, the available evidence remains heterogeneous. This narrative review aims to summarize molecular biomarkers of oxidative and nitrosative stress in atopic dermatitis and depression and to explore their shared biological pathways, with particular emphasis on the skin–brain axis.

## 2. Materials and Methods

This narrative review was based on a structured literature search conducted in March 2026 using the PubMed database, using the following terms: (“atopic dermatitis” OR eczema) AND (“oxidative stress” OR “nitrosative stress” OR ROS OR RNS OR “redox imbalance”) AND (biomarker* OR marker* OR malondialdehyde OR “lipid peroxidation” OR “4-HNE” OR “8-OHdG” OR “protein carbonyl*” OR AOPP OR “nitric oxide” OR iNOS OR nitrotyrosine OR glutathione OR “superoxide dismutase” OR catalase OR “antioxidant capacity”); (“depression” OR “major depressive disorder” OR MDD) AND (“oxidative stress” OR “nitrosative stress” OR ROS OR RNS OR “redox imbalance”) AND (biomarker* OR marker* OR malondialdehyde OR “lipid peroxidation” OR “4-HNE” OR “8-OHdG” OR “protein carbonyl*” OR AOPP OR “nitric oxide” OR iNOS OR nitrotyrosine OR glutathione OR “superoxide dismutase” OR catalase OR “antioxidant capacity”). The search yielded 238 records for AD and 2772 records for depression. Titles and abstracts were reviewed for relevance, and potentially relevant articles were assessed in full text. Studies investigating oxidative or nitrosative stress biomarkers in AD or depression were considered for inclusion. Only articles published in English were included. Case reports, conference abstracts, and studies without relevant biomarker data were excluded. Following full-text evaluation and additional selection based on relevance to the scope of this review, 90 articles were included for further analysis and discussion.

## 3. Results

### 3.1. Oxidative and Nitrosative Stress in Atopic Dermatitis

#### 3.1.1. Lipid Peroxidation in Atopic Dermatitis

Lipid peroxidation is a well-recognized feature of oxidative stress in atopic dermatitis. Its main products, particularly malondialdehyde (MDA) and 4-hydroxynonenal (4-HNE), reflect oxidative damage to membrane lipids and may contribute directly to inflammation and tissue injury [10,11]. MDA remains the most frequently assessed marker, although its interpretation is limited by small study populations and variability in biological samples [12]. Most human studies report increased lipid peroxidation in AD, with elevated MDA and other oxidative damage markers accompanied by reduced antioxidant defenses compared with healthy controls [13,14]. Consistent with this, recent reviews highlight MDA, 8-OHdG, and glutathione-related markers as among the biomarkers most closely associated with disease activity and severity, despite some variability between studies [5,15]. Experimental data further support the relevance of lipid peroxidation in AD. Mitochondrial dysfunction in the epidermis has been linked to accumulation of MDA, 4-HNE, and oxidized phosphatidylcholines, together with impaired glutathione-dependent antioxidant defense, indicating disturbed redox homeostasis at the cellular level [16]. Similar findings in keratinocyte models show that antioxidant interventions can reduce lipid hydroperoxides, nitric oxide, and inflammatory mediator expression [17,18]. From a clinical perspective, urinary biopyrrins have been shown to correlate with disease severity and inflammatory markers such as IgE (immunoglobulin E) and thymus and activation-regulated chemokine (TARC), suggesting potential utility in disease monitoring [19].

#### 3.1.2. Protein Oxidation in Atopic Dermatitis

Protein oxidation is another component of oxidative stress in atopic dermatitis, although it has been studied less extensively than lipid peroxidation. Advanced oxidation protein products (AOPPs) and protein carbonyls are commonly used markers of oxidative protein damage and have been linked to barrier dysfunction and disease activity [5,20]. Clinical studies generally report elevated AOPP levels in AD, indicating increased oxidative burden, although the differences may be less pronounced than in other inflammatory skin diseases [15,21]. In addition, increased protein carbonylation in the stratum corneum and lesional skin has been shown to correlate with xerosis and disease severity, suggesting a role in epidermal barrier impairment [22,23]. Although these markers are not yet used in routine clinical practice, they may complement other oxidative stress biomarkers in the assessment of AD pathophysiology [13].

#### 3.1.3. DNA Oxidation in Atopic Dermatitis

Oxidative DNA damage is an important aspect of redox imbalance in atopic dermatitis, with 8-OHdG being the most widely studied biomarker. Increased levels of 8-OHdG have been consistently observed in patients with AD, pointing to enhanced systemic oxidative stress [5,24]. Elevated urinary 8-OHdG, particularly in children, has been linked to disease risk, severity, and the presence of other atopic conditions such as asthma [24]. Environmental exposures, including ultrafine particles and polycyclic aromatic hydrocarbons, may further contribute to oxidative DNA damage, highlighting the role of external factors [25]. Although the available data are still limited, oxidative DNA damage appears to reflect systemic oxidative burden in AD and may offer additional insight into underlying disease mechanisms [11,15,26].

#### 3.1.4. Nitrosative Stress in Atopic Dermatitis

Nitrosative stress, driven by nitric oxide (NO) and reactive nitrogen species (RNS), is another component of redox imbalance in atopic dermatitis. Increased NO production has been reported in patients with atopic diseases, suggesting activation of the arginine-NO pathway [27]. Experimental studies show upregulation of inducible nitric oxide synthase (iNOS) and accumulation of nitrotyrosine in inflammatory skin lesions, pointing to enhanced protein nitration and local tissue damage [28,29]. These processes involve immune cells, including eosinophils, and contribute to inflammatory signaling. Although clinical data are still limited, available evidence suggests that nitrosative stress markers may reflect disease activity. This is supported by experimental models in which modulation of NO pathways reduces oxidative damage and inflammatory responses in keratinocytes [17,18].

#### 3.1.5. Antioxidant Defense Systems in Atopic Dermatitis

Impaired antioxidant defense is a consistent feature of oxidative stress in atopic dermatitis. Both enzymatic and non-enzymatic systems, including superoxide dismutase (SOD), catalase, glutathione, and glutathione peroxidase (GPx), appear to be altered, although findings vary across studies [5,16,20]. Experimental data indicate that mitochondrial oxidative stress in the AD epidermis is linked to impaired glutathione-dependent defense and reduced catalase and GPx activity, which may limit the ability of keratinocytes to adapt to oxidative injury [16]. Clinical studies likewise report reduced antioxidant capacity, altered thiol–disulfide homeostasis, and decreased total antioxidant status in both children and adults with AD, in some cases correlating with disease severity [13,15,30,31]. Additional evidence suggests that specific antioxidant-related pathways, including catalase activity, glutathione metabolism, and GST polymorphisms, may contribute to disease susceptibility and patient stratification [21,32]. In experimental models, antioxidant interventions restore enzyme activity, reduce ROS levels, and attenuate inflammatory signaling, supporting their potential therapeutic relevance [18,33,34].

### 3.2. Oxidative and Nitrosative Stress in Depression

#### 3.2.1. Lipid Peroxidation in Depression

Lipid peroxidation is one of the most consistently reported features of oxidative stress in major depressive disorder. Meta-analytic evidence shows increased levels of lipid peroxidation products, including malondialdehyde (MDA), 4-hydroxynonenal, peroxides, and 8-isoprostanes, together with reduced lipid-associated antioxidant defenses [35]. This pattern reflects a shift toward a pro-oxidative state linked to immune activation and neurotoxic effects. Consistent with this, large meta-analyses report elevated MDA and 8-isoprostanes alongside decreased total antioxidant capacity (TAC), with partial normalization following antidepressant treatment [36]. Clinical studies further support these findings, showing increased plasma MDA levels in MDD patients, in some cases with good diagnostic performance [37]. Elevated thiobarbituric acid reactive substances (TBARSs) have also been associated with immune activation and may reflect trait-related oxidative imbalance [38].

Other lipid peroxidation markers, such as 8-isoprostanes, are particularly increased in specific populations, including late-life depression, where they correlate with cognitive impairment [39]. Higher MDA levels have also been linked to symptom severity and may predict depressive outcomes in conditions such as post-stroke depression [40]. Increased lipid peroxidation appears to be a robust feature of depression, closely associated with reduced antioxidant defenses and immune-inflammatory dysregulation. This pattern parallels findings in atopic dermatitis, supporting the presence of shared redox-related mechanisms across inflammatory and neuropsychiatric disorders.

#### 3.2.2. Protein Oxidation in Depression

Protein oxidation and nitrosative stress are important components of redox imbalance in major depressive disorder, extending oxidative damage beyond lipids to structural and functional protein alterations. Increased levels of protein carbonyls and advanced oxidation protein products have been reported in depression and are associated with disease severity and broader nitro-oxidative stress profiles [41,42]. These findings point to a systemic disturbance involving both reactive oxygen and nitrogen species. Nitrosative pathways further contribute through dysregulation of the L-arginine–NO system and activation of NADPH oxidase, linking oxidative damage with vascular and inflammatory alterations [43]. At the molecular level, activation of redox-sensitive transcription factors such as NRF2 and NF-κB, together with altered antioxidant defenses, suggests an insufficient compensatory response to oxidative stress [44]. Together, these processes contribute to cellular dysfunction and neuroprogression and show clear overlap with inflammatory conditions such as atopic dermatitis.

#### 3.2.3. DNA Oxidation in Depression

Oxidative damage to nucleic acids represents another level of redox dysregulation in major depressive disorder. The most widely studied biomarker, 8-hydroxy-2′-deoxyguanosine (8-OHdG), is consistently elevated in patients with depression and has been linked to disease severity and recurrence [45,46]. Increased 8-OHdG levels have also been associated with the development of depressive symptoms in specific contexts, including post-stroke depression [47]. At the central level, increased DNA oxidation accompanied by upregulation of repair enzymes has been observed in brain tissue, suggesting an ongoing but insufficient compensatory response [48]. Oxidative damage also affects RNA, with elevated levels of oxidized RNA products reported in severe depression, indicating broader nucleic acid vulnerability [49]. Although findings are not entirely consistent, nucleic acid oxidation is generally considered a marker of chronic oxidative burden rather than a purely state-dependent feature [50]. These processes reflect sustained oxidative stress and may contribute to neuroprogression and accelerated biological aging, paralleling observations in inflammatory conditions.

#### 3.2.4. Nitrosative Stress in Depression

Nitrosative stress is an important component of oxidative–nitrosative imbalance in MDD and closely interacts with inflammatory signaling. Increased production of reactive nitrogen species, particularly through dysregulated NO pathways, leads to protein nitrosylation, lipid damage, and neuroinflammation. Elevated levels of markers such as 3-nitrotyrosine and NO metabolites, along with increased nitric oxide synthase activity, have been reported in patients with depression [51,52]. Similar findings in first-degree relatives suggest that nitrosative dysregulation may reflect trait vulnerability. At the molecular level, increased protein nitrosylation and immune responses to NO-adducts link nitrosative stress with inflammatory and autoimmune-like processes [53,54]. Excessive NO can also promote neurotoxicity, microglial activation, and cytokine release, further contributing to depressive pathophysiology [55]. Nitrosative stress integrates immune, metabolic, and neurobiological mechanisms and parallels redox disturbances observed in inflammatory conditions such as atopic dermatitis.

#### 3.2.5. Antioxidant Defense Systems in Depression

Disturbances in antioxidant defense are consistently reported in MDD and involve both enzymatic and non-enzymatic systems, including glutathione, superoxide dismutase, catalase, glutathione peroxidase, and total antioxidant capacity [56]. Reduced brain glutathione levels, demonstrated in neuroimaging studies, support impaired central antioxidant defense and may already be present early in the disease course [57,58]. Peripheral findings further indicate reduced antioxidant protection, including lower NRF2-related signaling and decreased antioxidant enzyme activity, accompanied by increased oxidative stress markers [59]. Decreased GPx activity and reduced total antioxidant capacity have been associated with greater oxidative burden and cognitive impairment, with partial normalization following antidepressant treatment [39,60]. Lower paraoxonase-1 activity also supports the presence of trait-related antioxidant dysfunction [61]. Impaired antioxidant defense appears to be a key feature of MDD pathophysiology and a potential therapeutic target.

### 3.3. Shared Oxidative and Nitrosative Pathways Linking Atopic Dermatitis and Depression

**Figure 1:**
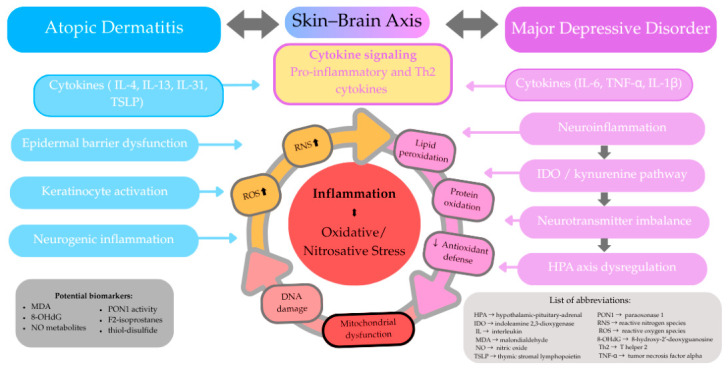
Schematic overview of shared inflammatory and oxidative–nitrosative mechanisms linking atopic dermatitis and depression within the skin–brain axis. Arrows indicate proposed directional associations between biological processes.

A schematic overview of the proposed shared inflammatory and oxidative–nitrosative pathways linking atopic dermatitis and depression within the skin–brain axis is presented in Figure 1.

#### 3.3.1. Inflammation and Redox Imbalance

A growing body of evidence suggests that atopic dermatitis and depression share a common biological pattern in which chronic inflammation and oxidative–nitrosative imbalance mutually reinforce one another. Rather than representing separate abnormalities, these processes appear to form a self-sustaining loop contributing to disease persistence and systemic consequences in both conditions. In AD, despite heterogeneity across studies, the overall evidence supports increased oxidative burden and an active role of redox imbalance in inflammatory skin injury [11]. Mechanistic studies support this view. Keratinocyte-derived reactive oxygen species activate pathways involved in type 2 inflammation, including KLK5, PAR2, NF-κB, TSLP, IL-25, and IL-33, while antioxidant treatment suppresses these responses, indicating that ROS function not only as by-products but also as upstream amplifiers of epithelial and immune activation [62]. Additional evidence points to broader epidermal redox disturbance, including mitochondrial stress, lipid peroxidation, and impaired glutathione-related defense, all associated with disrupted epidermal homeostasis [16]. Oxidative injury linked to TRPV3 activation further promotes keratinocyte damage and inflammatory signaling, supporting the pathogenic relevance of redox-dependent mechanisms in AD [63]. A similar inflammatory–redox interaction is observed in depression. Oxidative and nitrosative stress is now considered an increasingly recognized component of depressive pathophysiology, closely linked to immune activation, neuroprogression, and reduced antioxidant defenses [6,64]. Meta-analytic data confirm increased oxidative damage, including elevated 8-OHdG and F2-isoprostanes, indicating a measurable systemic redox burden [7]. Clinical studies in untreated MDD show positive correlations between oxidative stress markers and pro-inflammatory mediators such as IL-6 and the IL-6/IL-10 ratio. At the same time, reduced antioxidant protection, increased cytokine activity, DNA damage, and H_2_O_2_ generation support the presence of a self-perpetuating inflammatory–redox cycle [65]. Alterations in ROS/RNS parameters together with cortisol in drug-naïve patients further highlight the integration of inflammatory, redox, and stress-related pathways [52]. Taken together, these findings suggest that AD and depression converge on a shared mechanism in which persistent inflammation promotes oxidative and nitrosative stress, while redox imbalance further amplifies inflammatory signaling and tissue dysfunction. In AD, this loop is expressed mainly through barrier disruption, keratinocyte activation, and cutaneous immune responses, whereas in depression, it manifests through neuroimmune activation and systemic oxidative injury. This inflammatory–redox convergence may represent a key mechanism linking chronic skin inflammation with depressive vulnerability.

#### 3.3.2. Cytokines, Neuroimmune Signaling, and HPA Axis

Cytokine signaling and neuroimmune interactions represent a key interface linking inflammatory skin diseases and psychiatric disorders. In both AD and depression, immune activation extends beyond local inflammation and engages systemic pathways involving neuroendocrine regulation, oxidative stress, and stress-response systems. This network provides a biological framework for the “skin–brain axis,” in which peripheral inflammation can influence central nervous system function and vice versa.

In AD, epithelial-derived cytokines such as thymic stromal lymphopoietin (TSLP), interleukin-25 (IL-25), and interleukin-33 (IL-33) play a central role in initiating and amplifying type 2 immune responses. Released by keratinocytes in response to stressors, they link barrier disruption with immune activation and contribute to pruritus, neurogenic inflammation, and systemic signaling [4]. Additional data suggest involvement of interleukin-17(IL-17) and nitric oxide-related pathways, further connecting inflammatory signaling with redox imbalance [27,28,66]. Neuroendocrine regulation also appears relevant, as oxytocin signaling modulates inflammation and oxidative stress, while prenatal stress has been associated with increased AD risk, supporting a role for HPA axis-related mechanisms [67,68]. A comparable cytokine-driven neuroimmune network is well described in depression. Increased levels of pro-inflammatory cytokines, including IL-1, IL-6, and TNF-α, are associated with depressive symptoms and behavioral changes [69]. Recent proteomic evidence suggests that MDD may share Th2 skewing and dysregulation of immune- and neurovascular-related proteins with inflammatory skin diseases, including AD. He et al. further identified dupilumab, an IL-4Rα-targeting biologic that inhibits the Th2 axis, as potentially reversing several Th2-related inflammatory protein alterations in MDD in an in silico drug repurposing analysis [70]. Although these findings support the relevance of Th2-related immune pathways in a subset of MDD patients, Th2-targeted or oxidative/nitrosative stress-targeted therapy should currently be regarded as an emerging research direction that requires further investigation rather than an established treatment strategy. These cytokines influence neurotransmission partly through activation of the indoleamine 2,3-dioxygenase pathway, shifting tryptophan metabolism toward neurotoxic kynurenine metabolites and impairing serotonergic signaling [71]. Neuroimmune activation is also closely linked to HPA axis dysregulation, as altered cortisol and ACTH levels are associated with disease severity, cognitive dysfunction, and redox imbalance [52,72]. Early life stress further contributes to long-term changes in cytokine profiles and oxidative stress, supporting developmental programming of neuroimmune systems [73,74]. Gut microbiota dysbiosis may represent an additional shared factor linking AD, MDD, inflammation, and oxidative/nitrosative stress. Recent evidence suggests that neuropsychiatric disorders, including MDD, and inflammatory skin diseases, including AD, may share microbial alterations such as reduced short-chain fatty acid-producing taxa, altered gut barrier integrity, and increased immune activation [75]. These changes may promote systemic cytokine release and thereby contribute to ROS/RNS generation, mitochondrial dysfunction, and impaired antioxidant defense. Dysbiosis may act as an amplifier of immune-redox dysregulation within the gut–brain–skin axis. Taken together, these findings indicate that AD and depression share a common network of cytokine-driven neuroimmune signaling and HPA axis dysregulation, tightly linked to oxidative and nitrosative stress. In AD, this network manifests through epithelial cytokine release and stress-modulated immune responses, whereas in depression, it involves central immune activation, altered neurotransmission, and neuroendocrine imbalance. Despite these differences, both conditions converge on overlapping immune-redox-stress pathways, providing a mechanistic basis for their frequent co-occurrence.

#### 3.3.3. Mitochondrial Dysfunction and Antioxidant Failure

Mitochondrial dysfunction and impaired antioxidant defense represent another major point of convergence between atopic dermatitis and depression. In both disorders, chronic inflammation and oxidative–nitrosative stress are accompanied by disrupted mitochondrial homeostasis, reduced capacity to neutralize reactive species, and impaired adaptive redox responses. These disturbances likely contribute to disease persistence by promoting cellular injury, altered signaling, and reduced tissue resilience. In AD, oxidative stress is closely linked to mitochondrial dysfunction within the epidermis. Keratinocytes show increased mitochondrial oxidative stress, reflected by elevated reactive species and lipid peroxidation, together with impaired glutathione-dependent defense and reduced antioxidant enzyme activity [16]. Experimental data further support a causal role, as mitochondria-targeted antioxidants such as MitoQ reduce inflammation and restore epidermal homeostasis. Additional studies point to altered mitochondrial gene expression and increased circulating cell-free mitochondrial DNA, both associated with greater disease severity [76,77]. Antioxidant imbalance also appears to contribute to disease chronicity, with evidence of disturbed thiol–disulfide homeostasis and reduced antioxidant protection in more active AD. Mechanistically, pathways such as NRF2 signaling highlight the importance of endogenous antioxidant responses in limiting oxidative stress and barrier dysfunction [32,78]. A similar pattern is observed in depression, where mitochondrial dysfunction is increasingly recognized as a core feature of pathophysiology. Clinical and preclinical studies indicate abnormalities in mitochondrial biogenesis, bioenergetics, apoptosis-related signaling, and redox regulation, linking energy imbalance with oxidative injury and impaired neuroplasticity [79,80,81]. Antioxidant dysfunction, particularly involving glutathione pathways, is consistently reported. Neuroimaging studies show reduced brain glutathione levels in patients with MDD, along with disrupted coupling between glutathione and glutamatergic activity, suggesting a link between antioxidant depletion, neurotransmission, and symptom severity [57,82]. Reduced NRF2-related signaling and antioxidant enzyme activity further support impaired redox adaptation in depression [59]. These findings suggest that mitochondrial dysfunction and antioxidant failure are not isolated abnormalities of the skin or brain, but elements of a shared systemic vulnerability. In AD, this is reflected in impaired epidermal redox adaptation and barrier instability, whereas in depression, it manifests as altered bioenergetics, oxidative damage, and disrupted neurochemical homeostasis. The overlap—particularly in glutathione metabolism, NRF2-related defense, and stress-induced mitochondrial injury—highlights mitochondria and endogenous antioxidant systems as promising targets for integrated therapeutic strategies.

#### 3.3.4. Clinical Relevance of Shared Biomarkers

From a clinical perspective, the overlap between atopic dermatitis and depression is particularly relevant, as several oxidative and nitrosative stress markers recur in both disorders. The most consistent shared candidates include lipid peroxidation products, thiol–disulfide balance parameters, paraoxonase-related measures, nitrosative stress markers, and indices of oxidative DNA damage. Although none of these markers is suitable as a stand-alone diagnostic tool, together they support the concept of a measurable redox signature linking chronic inflammatory skin disease with depressive pathology. In AD, clinical studies indicate that paraoxonase dysfunction, reduced total antioxidant capacity, and increased lipid hydroperoxides reflect impaired antioxidant defense and ongoing oxidative injury [14]. Disturbances in thiol–disulfide homeostasis, particularly in pediatric populations, further suggest that redox imbalance may serve as a marker of both oxidative burden and disease chronicity [30,31]. Additional non-invasive markers, including urinary biopyrrins, nitrates, and exhaled 8-isoprostane, have been associated with disease severity and treatment response, supporting their potential utility in monitoring oxidative stress in AD [83,84,85,86]. A similar pattern is observed in depression. Meta-analytic evidence shows reduced total antioxidant capacity and paraoxonase-related activity alongside increased oxidative damage markers [36]. Among individual biomarkers, malondialdehyde, nitric oxide metabolites, 3-nitrotyrosine, and 8-isoprostanes have been most consistently associated with depressive pathology [37,51,87]. Thiol–disulfide imbalance and increased oxidative DNA damage further support the presence of systemic redox dysregulation in depression [46,88]. Shared biomarkers across AD and depression reflect overlapping inflammatory–redox processes despite differences in clinical presentation. However, their current application in routine practice remains limited by methodological heterogeneity, variability in biological samples, and lack of standardization. At present, these markers are best considered translational tools that may support risk stratification, disease monitoring, and mechanistic understanding, rather than established diagnostic indicators. To provide a clearer and more organized comparison of the evidence discussed above, Table 1 summarizes the main oxidative and nitrosative stress-related markers reported in AD and MDD. The table presents each marker or pathway according to its biological significance, reported findings in AD and MDD, and interpretative relevance.

Based on the available evidence summarized in Table 1, lipid peroxidation markers, particularly MDA and 8-isoprostanes, oxidative DNA damage marker 8-OHdG, and glutathione-related antioxidant defense are among the most frequently investigated and consistently discussed overlapping redox alterations in AD and MDD. These markers may therefore represent well-documented candidates for future quantitative synthesis aimed at determining their relative magnitude and clinical significance.

#### 3.3.5. Potential Temporal Links Between Atopic Dermatitis and Depression

The link between atopic dermatitis and depression is increasingly seen as more than simple comorbidity, and rather as a process that may develop over time through shared inflammatory and oxidative–nitrosative mechanisms. AD usually begins early in life and represents the first stage of the atopic march, driven by skin barrier dysfunction, type 2 inflammation, environmental factors, and oxidative stress [3]. This suggests that early and persistent immune and redox disturbances may contribute to long-term systemic vulnerability. Epidemiological data support this association. Individuals with atopic disorders have a significantly higher risk of depression, including major depressive disorder, independent of major confounders [8]. Although causality cannot be established, these findings point toward a shared immune-inflammatory and oxidative background. This association should be interpreted cautiously. A two-sample Mendelian randomization study by Baurecht et al. found no evidence that genetically instrumented AD causally increases the risk of broad depression, probable MDD, ICD-defined MDD, or anxiety. Therefore, the AD–MDD relationship may reflect shared vulnerability factors, disease severity, chronic itch, sleep disturbance, treatment burden, comorbid atopic conditions, or inflammatory–redox mechanisms rather than a simple direct causal pathway [89]. This is consistent with the concept of the skin–brain axis, linking peripheral inflammation with central nervous system changes [9]. This relationship may begin even before clinical disease becomes apparent. Prenatal maternal distress has been associated with increased AD risk in offspring, together with changes in oxidative stress markers, including reduced glutathione balance and altered glucocorticoid pathways [68]. This supports the idea of early-life programming of immune and neuroendocrine systems. Experimental data provide further support. Keratinocyte-derived reactive oxygen species can induce type 2 inflammatory pathways, whereas antioxidant treatment attenuates these responses [62]. In animal models, targeting oxidative and inflammatory pathways improves both skin inflammation and depressive-like behavior, along with normalization of neuroendocrine parameters [90]. 

## 4. Discussion

This review integrates evidence from dermatological and psychiatric research, highlighting consistent alterations in oxidative and nitrosative stress biomarkers across atopic dermatitis and depression, including lipid peroxidation products (e.g., MDA, 4-HNE), oxidative DNA damage (8-OHdG), and impaired antioxidant defenses [5,35,36]. By synthesizing findings from both fields, it provides a unified redox–inflammatory framework supporting the concept of a shared skin–brain axis. Several limitations should be acknowledged. The available evidence is highly heterogeneous, reflecting differences in study populations (children vs. adults), biological matrices (serum, urine, skin tissue), analytical methods, and disease severity, which substantially limits comparability and reproducibility [12,20]. Most evidence is derived from cross-sectional designs, precluding causal inference and making it unclear whether oxidative stress represents a cause, consequence, or epiphenomenon of disease processes [6,7]. Several commonly used biomarkers, such as malondialdehyde and TBARSs, lack specificity and are influenced by methodological variability, which complicates interpretation and limits comparability across studies [5,15]. In addition, variability in biological sampling and assay techniques may further contribute to inconsistent findings across studies. Potential confounders such as pharmacological treatment, lifestyle factors, and comorbidities are inconsistently controlled, particularly in studies of depression [42]. Another significant limitation is the lack of standardized quantitative comparisons of oxidative and nitrosative stress biomarkers across studies. Although many reports describe elevated or reduced levels of markers such as MDA, 8-OHdG, glutathione-related parameters, antioxidant enzymes, and nitrosative stress markers, the magnitude of these changes is difficult to compare because of differences in biological matrices, laboratory assays, units of measurement, disease severity, treatment status, and control groups. Consequently, this narrative review cannot determine which biomarkers exhibit the largest effect sizes or the greatest clinical significance. Future systematic reviews and meta-analyses should specifically address this gap by quantitatively assessing the magnitude of changes in individual biomarkers and identifying those with the most reproducible and clinically meaningful alterations in AD and MDD. Finally, the lack of standardized biomarker panels limits current clinical applicability despite promising translational potential.

## 5. Conclusions

Atopic dermatitis and major depressive disorder appear to share a convergent pathophysiological framework characterized by persistent inflammation and oxidative–nitrosative imbalance. Across both conditions, consistent alterations include increased lipid, protein, and DNA oxidation, elevated nitrosative stress markers, and impaired antioxidant defenses, particularly involving glutathione-dependent systems. These redox disturbances interact with immune activation, cytokine signaling, and HPA axis dysregulation, potentially forming a self-sustaining loop. Mitochondrial dysfunction further amplifies this process, contributing to cellular damage in both the skin and the central nervous system. The overlap in biomarkers supports the concept of a skin–brain axis linking chronic dermatological inflammation with neuropsychiatric outcomes. However, despite promising translational potential, current evidence is limited by heterogeneity and lack of standardization, preventing routine clinical application. Future research should focus on longitudinal and mechanistic studies to clarify causality and evaluate whether redox-targeted interventions can simultaneously improve dermatological and psychiatric outcomes.

## Figures and Tables

**Table 1:** Structured summary of oxidative and nitrosative stress-related markers discussed in AD and MDD.

Marker	Meaning	Findings in AD	Findings in MDD	Interpretation
MDA	Lipid peroxidation marker	Increased MDA/lipid peroxidation reported in AD	Increased MDA reported in MDD	Shared marker of lipid oxidative damage
8-OHdG	Oxidative DNA damage marker	Increased in AD, associated with severity/systemic burden	Increased in MDD, linked to severity/recurrence	Indicates systemic oxidative DNA damage
GSH	Major antioxidant redox buffer	Impaired glutathione-related defense in AD	Reduced glutathione-related protection in MDD	Shared impairment of antioxidant defense
SOD/CAT/GPx	Antioxidant enzymes	Altered activity reported, variable direction	Altered activity reported, variable direction	Suggests antioxidant dysregulation, but heterogeneous
AOPP/protein carbonyls	Protein oxidation markers	Increased in AD, linked to barrier dysfunction/xerosis	Increased in MDD, linked to nitro-oxidative profile	Protein-level oxidative injury
NO/iNOS	Nitrosative stress markers	Increased NO/iNOS/nitrotyrosine in inflammatory skin lesions/models	Altered NO pathways and nitrotyrosine-related markers in MDD	Shared nitrosative stress pathway
TAC	Global antioxidant capacity	Reduced antioxidant capacity/status in AD	Reduced antioxidant capacity in MDD	Global marker of reduced antioxidant protection
PON1	Lipid-associated antioxidant enzyme	Paraoxonase dysfunction reported in AD	Lower PON1-related activity reported in MDD	Lipid-associated antioxidant impairment
8-isoprostanes/F2-isoprostanes	Stable lipid peroxidation markers	Reported in AD, including non-invasive measurements	Increased in depression/meta-analytic evidence	Additional lipid peroxidation marker
Mitochondrial dysfunction	Source and amplifier of ROS	Epidermal mitochondrial stress in AD	Mitochondrial dysfunction linked to MDD pathophysiology	Shared amplifier of redox imbalance

## Data Availability

No new data were created or analyzed in this study. Data sharing is not applicable to this article.

## Abbreviations

The following abbreviations are used in this manuscript:

AD	Atopic dermatitis
AOPP	Advanced oxidation protein products
ACTH	Adrenocorticotropic hormone
DNA	Deoxyribonucleic acid
GSH	Glutathione
GPx	Glutathione peroxidase
HPA axis	Hypothalamic–pituitary–adrenal axis
IgE	Immunoglobulin E
iNOS	Inducible nitric oxide synthase
IL-1	Interleukin-1
IL-6	Interleukin-6
IL-17	Interleukin-17
IL-25	Interleukin-25
IL-33	Interleukin-33
MDD	Major depressive disorder
MDA	Malondialdehyde
NF-κB	Nuclear factor kappa B
NO	Nitric oxide
NRF2	Nuclear factor erythroid 2–related factor 2
PON1	Paraoxonase-1
RNS	Reactive nitrogen species
ROS	Reactive oxygen species
SOD	Superoxide dismutase
TSLP	Thymic stromal lymphopoietin
TARC	Thymus and activation-regulated chemokine
TBARS	Thiobarbituric acid reactive substances
TAC	Total antioxidant capacity
4-HNE	4-hydroxynonenal
8-OHdG	8-hydroxy-2′-deoxyguanosine
